# Major genes determining yield-related traits in wheat and barley

**DOI:** 10.1007/s00122-017-2880-x

**Published:** 2017-03-17

**Authors:** Anna Nadolska-Orczyk, Izabela K. Rajchel, Wacław Orczyk, Sebastian Gasparis

**Affiliations:** 1 Department of Functional Genomics, Plant Breeding and Acclimatization Institute - National Research Institute, Radzikow, 05-870 Blonie, Poland; 2 Department of Genetic Engineering, Plant Breeding and Acclimatization Institute - National Research Institute, Radzikow, 05-870 Blonie, Poland

## Abstract

**Key message:**

Current development of advanced biotechnology tools allows us to characterize the role of key genes in plant productivity. The implementation of this knowledge in breeding strategies might accelerate the progress in obtaining high-yielding cultivars.

****Abstract**:**

The achievements of the Green Revolution were based on a specific plant ideotype, determined by a single gene involved in gibberellin signaling or metabolism. Compared with the 1950s, an enormous increase in our knowledge about the biological basis of plant productivity has opened new avenues for novel breeding strategies. The large and complex genomes of diploid barley and hexaploid wheat represent a great challenge, but they also offer a large reservoir of genes that can be targeted for breeding. We summarize examples of productivity-related genes/mutants in wheat and barley, identified or characterized by means of modern biology. The genes are classified functionally into several groups, including the following: (1) transcription factors, regulating spike development, which mainly affect grain number; (2) genes involved in metabolism or signaling of growth regulators—cytokinins, gibberellins, and brassinosteroids—which control plant architecture and in consequence stem hardiness and grain yield; (3) genes determining cell division and proliferation mainly impacting grain size; (4) floral regulators influencing inflorescence architecture and in consequence seed number; and (5) genes involved in carbohydrate metabolism having an impact on plant architecture and grain yield. The implementation of selected genes in breeding programs is discussed, considering specific genotypes, agronomic and climate conditions, and taking into account that many of the genes are members of multigene families.

**Electronic supplementary material:**

The online version of this article (doi:10.1007/s00122-017-2880-x) contains supplementary material, which is available to authorized users.

## Introduction

Allohexaploid bread wheat (*Triticum aestivum* L., 2*n* = 6*x* = 42, AABBDD) and diploid barley (*Hordeum vulgare*) are very important crops in global agriculture. Wheat ranks third after maize and rice, and barley is the fourth (Schulte et al. 2009; Tester and Langridge 2010). As temperate crops, their production in EU countries is especially high.

The harvested production of wheat and barley in 2014 was 149.7 and 60.8 million metric tons, which makes up, respectively, 44.8 and 18.2% of the total cereal production in the EU (http://ec.europa.eu/eurostat/statistics-explained/index.php/Agricultural_production_-_crops). The species are major crops in the Triticeae tribe. Because of their close relationship, they share common traits, which are distinct from rice and maize. The Triticeae grown worldwide are well adapted to a temperate climate, have winter and spring types, and have similar architecture of inflorescences and spikes. Winter varieties, in contrast to spring type, require vernalization to promote flowering in response to long-day (LD) conditions.

The Green Revolution (GR) period, which was initiated in the 1940s and continued until the late 1960s, resulted in a significant increase in cereal production mainly due to higher yields. It was achieved by combining natural semi-dwarf mutants with specifically changed plant architecture and improved agrotechnology including irrigation and fertilization. Further research revealed that the most important traits were caused by single gene mutations: *Reduced height-1 (Rht1*) in wheat and *semidwarf1* (*sdw1*) in barley (reviewed in Hedden 2003). The genes and the mutations affecting gibberellin signaling and metabolism led to pleiotropic changes which were beneficial for crop production (Börner et al. 1993; Chandler and Harding 2013; Franckowiak and Lundqvist 2012; Peng et al. 1999; Saville et al. 2012; Wen et al. 2013).

Currently, bred varieties are still characterized by higher yields, but the increase has never been as spectacular as that observed during the GR. An important limitation is related to the large and complex genomes of both crops, which makes selection of crucial traits particularly difficult. However, continuing from the 1970s, a significant increase of knowledge on cereal genomes and gene functions as well as development of new biotechnological tools has opened new possibilities to repeat the success of the GR. The knowledge on a particular gene’s function in relation to grain yield is especially important for efficient selection of new haplotypes in classical breeding. This can be a new driver of wheat and barley breeding.

Several reviews on genetic factors influencing various aspects of cereal yield have already been published. There are articles on: genetic factors of floral development and inflorescence architecture in cereals (Sreenivasulu and Schnurbusch 2012), control of flowering time (Cockram et al. 2007), aspects of senescence, nutrient remobilization and yield in wheat and barley (Distelfeld et al. 2014), traits associated with phenology, photosynthesis, assimilate partitioning, and lodging resistance in rice and wheat (Valluru et al. 2014), genetic factors improving barley culm robustness (reviewed by Dockter and Hansson 2015), and genes regulating cytokinin levels in various species (reviewed by Jameson and Song 2015).

In our review, we focus on the most important and newly identified genes determining yield-related traits in the Triticeae species wheat and barley. The selected genes represent different groups with various biological functions. We start with those that were so successfully used during the GR but thoroughly characterized in the last decade, and then continue with the newly identified genes. We also indicate the sources and the types of gene modifications (mutants, transgenics, positional cloning, etc) and discuss how the changes affect yield components.

## Plant architecture and grain yield traits in Triticeae

Barley and wheat belong to the Triticeae species possessing similar plant architecture with an unbranched inflorescence known as the spike. The architecture as well as their climatic and agronomic requirements differ from the model species rice. Spikes of wheat and barley most likely evolved from an ancestral compound inflorescence, producing branches (Endress 2010; Kellogg et al. 2013; Remizowa et al. 2013). Archetypal inflorescences and spikes bear three single-flowered spikelets per rachis node. The spikelets are arranged in two opposite rows along the main axis. In barley, if the two outer lateral spikelets at each node are sterile, the spikes are two-rowed; when all three are fertile, the spikes are six-rowed (Ramsay et al. 2011). Final grain yield depends on grain number and grain weight (reviewed by Sreenivasulu and Schnurbusch 2012; Kesavan et al. 2013; Distelfeld et al. 2014). The yield components influencing grain number include number of tillers bearing fertile spikes, extension of vegetative as well as reproductive growth and differentiation phase, inflorescence architecture, culm hardiness, spike initiation, elongation and branching as well as spikelet formation. Grain weight is affected by grain cell number (Brocklehurst 1977) and sink capacity (Millet and Pinthus 1984).

## The genomes of barley and wheat

The size and the complexity of barley and bread wheat genomes are very important obstacles in developing different strategies of selection and breeding. The size of the barley (*Hordeum vulgare*) genome is 5.1 Gbp (Doležel et al. 1989), and that of wheat (*Triticum aestivum*) is over three times bigger, 17 Gbp (Bennett and Smith 1976; The International Wheat Genome Sequencing 2014). The two genomes also differ considerably with respect to their complexity. Barley is diploid, containing seven chromosome pairs (2*n* = 2*x* = 14). Wheat is allohexaploid (AABBDD), with three diploid genomes originating from different species (2*n* = 6*x* = 42). More than 124,000 gene loci were annotated by The International Wheat Genome Sequencing Consortium (2014). The genes are distributed in gene-rich regions at the telomeres across the homoeologous chromosomes and subgenomes (Gill et al. 2006a, b). The data gathered by the consortium evidenced dynamic genome change including gene gain, loss, and duplication since the divergence of wheat lineages.

Due to the allohexaploidy, the majority of wheat genes are represented by three homologs in each of the three A, B, and D genomes. However, considering their effect on phenotype, the most important question is whether they are expressed and, if so, what their expression pattern is. Data collected so far indicate that only about 20% of genes were always expressed from all three genomes (Mochida et al. 2003). The remaining ones showed preferential expression from certain genomes, which could vary between tissues and organs (Appleford et al. 2006; Mochida et al. 2003). Analysis of the wheat grain transcriptome revealed cell type and stage-dependent genome dominance and asymmetric expression for some groups of genes (Pfeifer et al. 2014). There are only a few examples of wheat genes that are located exclusively in one genome, such as *Pin* genes, which determine grain hardness (Nadolska-Orczyk et al. 2009).

## The genes of the Green Revolution

The Green Revolution (GR) and its founder Norman Borlaug are widely recognized iconic symbols of the 20th century agriculture. The novel approach to breeding crop varieties and a significant increase in productivity crucial to meet food demand in hunger-threatened regions were recognized by awarding the 1970 Nobel Peace Prize to Norman Borlaug. The goal was achieved by the development of high-yielding varieties together with the application of modern agrotechnologies, such as irrigation, synthetic fertilizers and pesticides (reviewed by Hedden 2003).

These high-yielding varieties, which were the key to success, were developed by the introduction of single major genes into cereals. The new varieties were shorter, with improved resistance to stem lodging and greater ability to tolerate nitrogen-based fertilizers (Gale and Youssefian 1985). Moreover, due to photoperiod insensitivity and rust resistance, the cultivars were adapted to a wide range of agricultural environments (Borlaug 1983).

Semi-dwarf phenotype is the most important feature of GR cultivars, and in wheat, it depends on the presence of the *Reduced height-1* (*Rht1*) gene. The gene was identified in Japan after crossing a Japanese semidwarf wheat cultivar with American high-yielding varieties to produce Norin 10 (reviewed by Hedden 2003). Two alleles of the gene are located at one of two loci on genome B and genome D of hexaploid wheat and are named, respectively, *Rht-B1* and *Rht-D1* (Börner et al. 1996). They have been found in the majority of varieties grown worldwide (Evans 1998). The alleles confer a limited response to the growth phytohormone gibberellin (GA) (Gale and Marshall 1973). Further research revealed that the *Rht* alleles encode DELLA proteins (Peng et al. 1999), which are transcriptional regulators that act to repress GA signaling (Pearce et al. 2011). The mutated alleles contain single nucleotide substitutions that determine premature stop codons in the N-terminal coding region (Peng et al. 1999). *Rht-B1* and *Rht-D1* are orthologs of the *Arabidopsis Gibberellin Insensitive* (*GAI*) gene and maize *dwarf-8* (*d8*) (Chandler et al. 2002; Peng et al. 1999) as well as barley *Slender1* (*HvSln1*) (Wen et al. 2013). The direct effect of the mutation is reduced plant height, but there is also an important pleiotropic effect causing increased assimilate partitioning to developing ears and an increased number of grains per spike (Börner et al. 1993; Flintham et al. 1997; Youssefian et al. 1992). In addition, selected alleles of the *Rht* gene play a role in disease resistance (Saville et al. 2012; Srinivasachary et al. 2009).

In barley, the Green Revolution genes *semidwarf1* (*sdw1*) located at the *sdw1*/*denso* locus (Druka et al. 2011; Franckowiak and Lundqvist 2012) and *uzu1.a* (Chono et al. 2003; Dockter et al. 2014) are involved, respectively, in the metabolism of the GA and brassinosteroid hormones. The genes determine shorter and stronger culms supporting spikes and preventing lodging. Since both phytohormones control many processes in different plant tissues, their pleiotropic effects also cause a number of unwanted agronomic traits, such as reduction of grain size. Moreover, *sdw1* confers late flowering and might be associated with lowered malt quality, while *uzu1* determines temperature sensitivity, and therefore, there were attempts to replace them with alleles of alternative genes (Dockter and Hansson 2015; Wang et al. 2014a).

The pleiotropic effect of another GR dwarfing gene of barley, *Sln1* mentioned above, include, similar to their orthologous *Rht* genes of wheat, disease resistance (Saville et al. 2012), which should be appreciated in plant breeding.

## Approaches for the identification of major yield determining genes

Natural or induced mutations were the only source of agricultural important traits until the end of the 1970s. Diploid barley has been, since the early 20th century, a model for obtaining induced mutants and exploring the potential of mutation breeding in crop improvement (Druka et al. 2011). The large mutant resources are collected in the NordGen genebank (http://www.nordgen.org/), and are proposed to be explored using the tools of modern genetics. Barley mutants have been used to characterize and/or utilize genes of nitrate reductase (Somers et al. 1983), the row-type gene, *SIX-ROWED SPIKE 1* (*Vrs1*; Komatsuda et al. 2007), short culm *UZU DWARF* (*Uzu1*; Chono et al. 2003), and semi-dwarf, slender-type plant architecture *SLENDER1* (*Sln1*; Chandler et al. 2002). The nature of some morphological mutants of barley was proposed to be explained by a new model of barley phytomer (Forster et al. 2007). Selection of such mutants is especially difficult in the complex, polyploid wheat genome.

Since the 1980s, more sophisticated approaches to acquire new phenotypes for research and breeding have been developed. The methods include genetic transformation leading to transgene or native gene overexpression or native gene silencing. RNAi-based silencing of a selected gene is currently the most powerful technology among genetic modification methods, especially useful for analysis of gene function in species with large and complex genomes including wheat (reviewed in Fu et al. 2007; Gasparis et al. 2011, 2013; Lawrence and Pikaard 2003; Travella et al. 2006).

The availability of molecular marker-based genetic maps allowed detection of hundreds of yield-related quantitative trait loci (QTL), and, in species with smaller genomes, e.g., rice, multiple QTL have been cloned (Mayer et al. 2011). Direct cloning of yield-related genes using strategies of gene high-resolution mapping and map-based cloning in wheat and barley is still very difficult due to the complexity of the genomes. However, availability of the sequenced genomes and transcriptomes as well as the development of comparative genomics creates new possibilities of gene identification and gene function analysis (Christiansen et al. 2016; Hou et al. 2014; Ma et al. 2015a; Poursarebani et al. 2015; Qu et al. 2015; Vu et al. 2010a, b).

The most impressive progress has been made for rice, a species with a relatively small, fully sequenced and annotated genome, which has become a model cereal species. The Triticeae species differ from rice with respect to their plant architecture including inflorescence as well as their climatic and agronomic requirements. It is worth noting that phenotype differences found for rice and Triticeae correspond to differences and relationships between groups of major genes. However, despite the differences, some yield-related genes in wheat and barley have been identified using comparative genomics with rice (Distelfeld et al. 2012; Hanif et al. 2016; Houston et al. 2013; Hu et al. 2016; Jiang et al. 2011; Ma et al. 2015a, Su et al. 2011; Zheng et al. 2014) or other species including the model plant *Arabidopsis* (Jost et al. 2016). Typically, the identified orthologs retained the same biological function, although in certain cases, they might determine different phenotypes (Distelfeld et al. 2012; Gasparis et al. 2013). This shows that a particular function of a gene of interest should be established in a target species. Some information on expected function might be derived from the temporal and spatial expression profile of selected genes (Christiansen et al. 2011; Ma et al. 2015b; Song et al. 2012; Zalewski et al. 2014).

Currently, genome editing is expected to be the most powerful biotechnology tool to perform specific changes through targeted mutagenesis, precise gene editing, multigene transformation, and gene stacking. The technology will allow for gene inactivation or generation of a new functional allele. There are already the first papers on induction of targeted, heritable mutations in barley, and *Brassica oleracea* (Lawrenson et al. 2015) and gene replacement in barley (Watanabe et al. 2015). Moreover, a group of researchers from the Chinese Academy of Sciences showed that TALEN and CRISPR-Cas9 technologies have been efficient and specific in allopolyploid species such as common wheat (*T. aestivum*), with triplicate homoeologous genes of the three genomes (Shan et al. 2014; Wang et al. 2014a, b). Application of the strategies to yield-related traits will require further technical improvement, adaptation to different genotypes and documentation of heritability. The gene silencing approach by RNAi-based technology might remain the method of choice in polyploid species, although the potential big advantage of genome editing tools is the possibility to obtain plants that might be excluded from GMO legislation.

## The major genes of the second GR

### Barley (*Hordeum vulgare*) genes and wheat (*Triticum aestivum, T. durum*) orthologs

Barley, with its smaller and less complex, compared to wheat, diploid genome, is more suitable for identification of natural or induced mutants, which result in higher yield. Among the 20 genes listed in Table S1, 18 are natural or induced mutants, and 2 genes were modified by RNAi-based silencing. The genes are classified functionally into three general groups (Table S1). The first one (I), associated with higher grain number, includes transcription factors and other regulators affecting spikelet development, inflorescence architecture and growth. Group II, controlling plant architecture, contains genes that directly affect metabolic processes or signaling of growth regulators. The genes changing cytokinin metabolism have an important influence on grain number; the genes modifying gibberellin and brassinosteroid metabolism mainly influence culm (stem) length and robustness/hardiness. Other mutants, frequently of unknown function, control spike and spikelet characteristics, awn and culm length and robustness (reviewed by Dockter and Hansson 2015). For some of the barley genes listed in Table S1, orthologs in wheat (Table S2) were found, and in such a situation, the genes of both species are described together. A list of barley and wheat yield-determining genes with known orthologs in other cereals/species is presented in Table 1.

**Table 1:** List of barley and wheat yield-determining genes with known orthologs in other cereals/species

Barley gene/synonym (localization)	Rice	Maize	Wheat	Others
*Vrs1* (2H)^1^	−	−	−	−
*Vrs4*/*HvRA2* ^4^	*Osra2* ^5^	*Zmra2* ^5^	*Tara2* ^5^	*ASL4* (*A. thaliana*)
*INT-C*/*HvTB1* (4H)^2^	*OsTB1*	*TB1* ^3^	−	*AtBRC1*
*Ppd-H1*	*OsPRR37* ^6^	−	*Ppd-D1* (2D)^7^	*AtPRR7* ^6^
*COM2*	*FZP*/*BFL1*	*BD1*	*bh* ^*t*^ (tetraploid), *WFZP*	−
*HvAP2* (2H)^8^	*OsEATB* ^9^	*dil1* ^10^		
*HvCKX* (family)	*OsCKX*	*ZmCKX*	*TaCKX*	+
*Sdw1*/*Denso* (3H)^12^	*SD1*	−	−	*AtGA5* ^11^
*HvSLN1*	*SLR1* ^13^	*d8* ^13^	*Rht-B1* (4B)^15^, *Rht-D1* (4D) ^5^	*AtGAI* ^13^
*Grd5*/*HvKAO1* ^14^	−	*Dwarf3*	−	−
*Uzu1* (3H)^12^	*D61*	−	−	−
*PcG*	*OsFIVE2* ^16^	−	−	−

Wheat	Rice	Maize	Barley	Others
*TaCKX6-D1*	*OsCKX2*	+	+	+
*TaTEF-7A*	*OsTEF1*	−	−	−
*TaGW2* (6A, 6B)	*OsGW2*	−	−	−
*TaTGW6-A1* (4A)^17^	*OsTGW6*	−	−	−
*TaGS5-3A-T*	*OsGS5*	−	−	−
*TaGS-D1*	*OsGS3*	−	−	−
*TaSus1* (7A), *TaSus2* (2A, 2B, 2D)^18^	*OsSUS*	*ZmSUS1*	*HvSUS*	*AtSUS*
*NFYAs, NFYBs, NFYCs* (families)	*OsNF-Y*	*ZmNF-YB2*	*HvNF*-Y	*AtNF*-Y
*TaNAC2*-5A	*OsNAC* ^19^	+	*HvNAC* ^20^	+
*TaCWI*	+^21^			

− Not known/not found

+ Possible orthologs (conserved protein among grasses)

Supplemental references: ^1^Komatsuda et al. (2007), ^2^Lundqvist et al. (1997), ^3^Ramsay et al. (2011), ^4^Koppolu et al. (2013), ^5^Bortiri et al. (2006), ^6^Higgins et al. (2010), ^7^Beales et al. (2007), ^8^Houston et al. (2013), ^9^Qi et al. (2011), ^10^Jiang et al. (2012), ^11^Barboza et al. (2013), ^12^Li et al. (2015), ^13^Wen et al. (2013), ^14^Jia et al. (2009), ^15^Börner et al. (1997), ^16^Nallamilli et al. (2013), ^17^Hu et al. (2016), ^18^Jiang et al. (2011), ^19^Distelfeld et al. (2012), ^20^Christiansen et al. (2011), ^21^Cho et al. (2005)

#### Transcription factors

A group of transcription factors regulate inflorescence architecture and spikelet development. Although mutants of some of them were identified decades ago, the detailed molecular analysis of selected alleles has been carried out during the last few years.

**Fig. 1:**
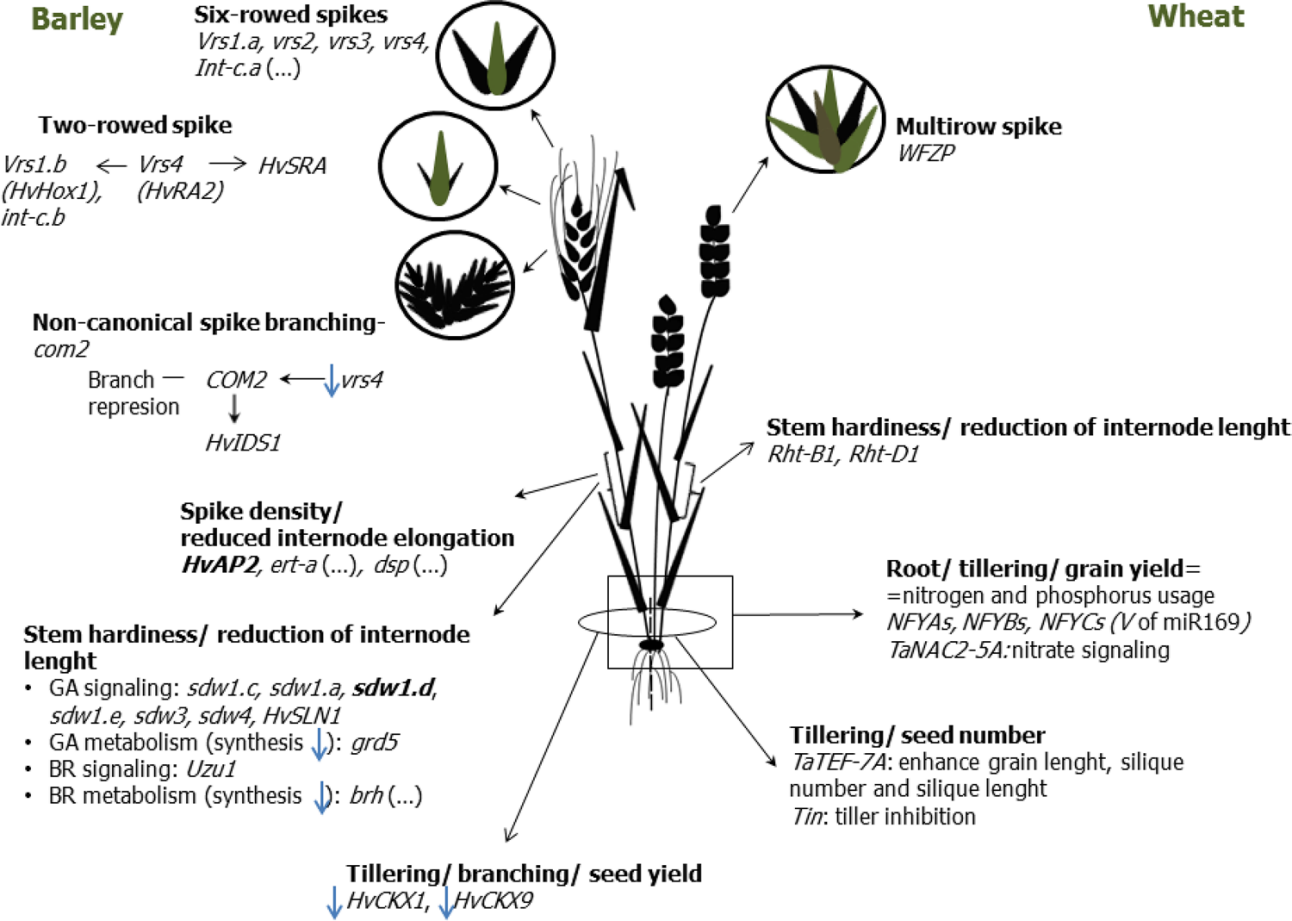
Major genes determining plant architecture in barley (*left*) and wheat (*right*). *Vertical arrows* indicate down or up enzyme regulation/gene expression; *horizontal arrows* indicate direction of gene co-regulation; *bold* main/key allele; *ellipsis* multiple alleles

The main genes modifying spikelet development by regulation of lateral spikelet fertility, *SIX-ROWED SPIKE 1* (*VRS1*) (Komatsuda et al. 2007; Lundqvist et al. 1997; Lundqvist and Lundqvist 1988) and *INTERMEDIUM-C* (*INT-C)* (Lundqvist and Lundqvist 1988; Ramsay et al. 2011), were found as natural mutants. Modifications of the genes in barley have resulted in two different cultivation types, two-rowed and six-rowed forms. Both forms are known and their origin is dated to the domestication period (Ramsay et al. 2011). A schematic diagram of relationship between these and other major genes determining plant architecture in barley and wheat is presented in Fig. 1.

*vrs1* is a loss-of-function allele, derived from the wild two-rowed ancestor. The wild-type allele *Vrs1.b* encodes the homeodomain-leucine zipper I-class (HD-Zip I) type transcriptional repressor that inhibits the development of lateral spikelet fertility (Komatsuda et al. 2007). The protein controls cell division and development specifically in the lateral spikelets. The *vrs1* loss-of-function allele determines the development of six-row spikes. The pleiotropic effect of the allele is the reduction in the number of tillers per plant, and thus spikes per plant, that is compensated for by the increase in number of grains per spike (Lundqvist et al. 1997). *Vrs1* orthologs of wheat, *Brachypodium*, rice, sorghum, and maize were not found, indicating that this transcription factor is specific to barley (Sakuma et al. 2010).

Phenotypes determined by *Vrs1* alleles are modified by alleles at the *INTERMEDIUM-C* (*INT-C*) locus (Ramsay et al. 2011). Lundqvist and Lundqvist (1988) found up to ten *INT* alleles. Seventeen additional barley mutations of the *INT-C* coding region correlating with lateral spikelet fertility were identified (Ramsay et al. 2011). The alleles influence variations in male fertility and grain development from two-rowed to six-rowed spikes. *INT-C* is an ortholog of the maize domestication gene *TEOSINTE BRANCHED 1* (*TB1*). Besides *Vrs1* and *Int-C, Six-rowed spike4* (*Vrs4*) is the third locus found to control row type in barley (Koppolu et al. 2013). *Vrs4* is an ortholog of the maize inflorescence architecture gene *RAMOSA2* (*RA2*), which encodes a lateral organ boundaries (Lima Neto et al. 2014) domain transcription factor. Eighteen coding mutations of the gene were associated with lateral spikelet fertility and loss of spikelet determinacy (Koppolu et al. 2013).

*Photoperiod-*1 (*Ppd-H1*) is the major determinant of barley photoperiod response (Turner et al. 2005). The *ppd1-H1* late-flowering mutant belongs to a class of genes involved in circadian clock function (Turner et al. 2005). The allele shows a reduced response to photoperiod, which is explained by altered circadian expression of the photoperiod pathway gene *CONSTANS* (*CO*) and reduced expression of its down-stream target *FLOWERING LOCUS* (*FT*), a key regulator of flowering (Turner et al. 2005). Both *FT1* and *Ppd-H1* have not been implicated in the control of two- or six-rowed spikes. However, the genes regulate optimum flowering time, specific for geographical regions, and essential for enhanced barley productivity. The latest research on *CONSTANS* genes in barley, *HvCO1* and *HvCO2*, showed that the genes control floral repression by upregulating *VERNALIZATION 2* (*VRN-H2*) (Mulki and Korff 2016).

Comparative mapping shows *Ppd-H1* of barley to be collinear with a wheat member of the pseudo-response regulator (*PRR*) gene family, *Ppd-D1* located on chromosome 2D (Beales et al. 2007). The *Ppd-1* gene in wheat, like in barley, is a key regulator of inflorescence architecture and paired spikelet development (Boden et al. 2015). The gene controls photoperiod-dependent floral induction by regulating expression of *FT*. Loss-of-function alleles promote a constitutive long-day (LD) response (Beales et al. 2007; Shaw et al. 2012, 2013; Turner et al. 2005). Among five *Ppd-D1* mutants found by Beales et al. (2007), only the *Ppd-D1a* allele was associated with photoperiod insensitivity. This loss-of-function allele has a 2 kb deletion upstream of the coding region and determines early flowering on short or long days. According to the authors (Beales et al. 2007), this type of photoperiod-insensitive wheat mutant allowed adaptation of cultivars obtained during the GR to broad range of environments. The *Ppd-1* photoperiod insensitive mutations were found in A, B, and D genomes, but there was no genome specificity associated with these mutants (Shaw et al. 2012). The flowering-affecting alleles act mostly by upregulation of *TaFT1* and suppression of *CONSTANS TaCO1*.

**Fig. 2:**
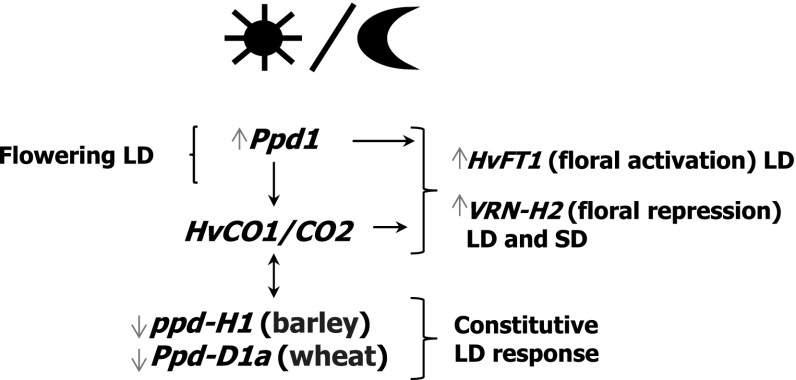
Major genes controlling photoperiod in barley and wheat. For the precise model of coregulation of these genes before and after vernalization, see Mulki and von Korff (2016)

Most of the genes described above have already been used in breeding. The breeding target proposed by Boden et al. (2015) will be the modulation of the expression of the two genes *Ppd-1* and *FT* to obtain wheat inflorescence showing better arrangements and an increased number of grains in spikelets. Moreover, reduced photoperiod responsiveness of the *ppd-H1* mutant of barley is considered to be advantageous in spring varieties (Turner et al. 2005). Regulation of interactions between *HvCO* and *PpdH1* with *VRN-H2* is proposed for manipulation of the photoperiod response together with the vernalization pathway in barley (Mulki and Korff 2016). A diagram of the potential relationship between the genes controlling the photoperiod in barley and wheat is presented in Fig. 2. Precise models for the coregulation of these genes in the SD or LD light (Cockram et al. 2007) and before and after vernalization were recently proposed by Mulki and von Korff (2016).

An induced mutant of inflorescence architecture named ‘Compositum-Barley’ displays noncanonical spike branching (Poursarebani et al. 2015). The spikelets are replaced by lateral branch-like structures forming small secondary spikes. The phenotype is determined by the *compositum 2* (*com2*) locus. *COM2* was found to be orthologous to the *branched head* ^*t*^ (*bh* ^*t*^) locus regulating spike branching in tetraploid ‘Miracle-Wheat’, and also orthologous to maize *BRANCHED SILKLESS* (*BD1*), to rice *FRIZZY PANICLE*/*BRANCHED FLORETLESS* 1 (*FZP*/*BFL1*) (Poursarebani et al. 2015) and to *WFZP* (*W* from wheat) in hexaploid wheat (Dobrovolskaya et al. 2015). *FRIZZY PANICLE* (*FZP*) is the main gene acting in spikelet development during the floret meristem transition phase. The orthologs encode a number of the APETALA2/Ethylene Response Factor transcription factor family members. The *COM2* expression in barley was found to be regulated by *Six-rowed spike 4* influencing branch repression in barley, while *HvIDS1* (an ortholog of maize *INDETERMINATE SPIKELET 1*) is a putative down-stream target of *COM2*. Wheat mutants developing supernumerary spikelets (SSs) are a recessive type of *FZP*/*BFL1*, and three *WFZP* homoeologous genes were found. The most severe effect was observed in genotypes sharing *WFZP-D* lesions and *WFZP-A* frameshift. The final result of noncanonical branch formation in tetra- or hexaploid wheat was production of significantly more grains per spike and higher yield. Therefore, supernumerary spikelet (SS) mutants are proposed to be included in wheat breeding to increase grain number (Dobrovolskaya et al. 2015; Poursarebani et al. 2015). Moreover, spike branching is expected to enhance sink capacity in wheat spikes (Lawlor and Paul 2014), which might further increase yield. Otherwise, the same mutation in branch repressor genes of barley and other diploid grass species did not influence grain number (Poursarebani et al. 2015).

A genome-wide analysis of common wheat identified seven QTL regulating supernumerary spikelet SS formation located on five chromosomes (2D, 5B, 6A, 6B, and 7B) (Echeverry-Solarte et al. 2014). Sequence analysis of the *bh* ^*t*^ locus in a collection of mutant and wild-type tetraploid wheat accessions revealed that a single amino acid substitution in the DNA-binding domain of the encoded protein gave rise to ‘Miracle-Wheat’ (Poursarebani et al. 2015).

The family of NAC transcription factors is known to be involved in regulation of important agronomic traits in plants. Comparison of selected barley *NAC* genes with closely related *NAC* genes from other species suggests their conserved role in cell wall biosynthesis, leaf senescence, root development, seed development, nutrient remobilization, and hormone-regulated stress responses (Christiansen et al. 2011). However, the functions for some *NAC* orthologs in wheat and rice were divergent (Distelfeld et al. 2012), which underlines the importance of separate functional characterization for each gene in a target species. Analyzed by comparative genomics, barley *HvNAC005* was shown to be a strong positive regulator of senescence (Christiansen et al. 2016). The authors indicate the gene as a target for tuning the gene expression to improve nutrient remobilization related to senescence in barley.

In wheat, the most prominent example of *NAC* is *NAM-B1* located at the *Gpc-B1* locus (Uauy et al. 2006a). The wild allele encodes a NAC transcription factor that accelerates senescence and improves grain protein, zinc, and iron content. Contrary silencing of expression of multiple *NAM* homologs by RNAi delayed senescence and significantly reduced protein, zinc, and iron in the grains. *Gpc-B1* is considered as a domestication gene affecting grain size (Dubcovsky and Dvorak 2007; Uauy et al. 2006b). Modern wheat varieties carry a nonfunctional *GPC-B1*. The authors suggest that this is a result of selection by breeders of larger seeds, which are the result of non-accelerated grain maturity.

Another nitrate-signaling and cereal-specific NAC transcription factor, *TaNAC2-5A*, was isolated and re-introduced into wheat to study nitrate-dependent signaling (He et al. 2015). Overexpression of the gene enhanced root growth and the nitrate influx rate, and as a consequence increased the root’s ability to acquire nitrate from the soil. Transgenic wheat lines revealed higher grain yield and higher nitrogen accumulation in aerial parts, which was allocated to grains. *TaNAC2-5A* was found to be involved in nitrate signaling and is proposed as a gene resource for breeding crops with more efficient use of fertilizers (He et al. 2015).

Barley spike density has been shown to be controlled by several loci: *erectoides-a* (*ert-a*), mapped to the centromere of chromosome 7H *dense spike* (*dsp*) (Shahinnia et al. 2012), and *ZEOCRITON1 Zeo1, Zeo2, Zeo3* (Druka et al. 2011), with the most important alleles of *HvAPETALA2* (*HvAP2*) interacting with microRNA172 (miR172) (Houston et al. 2013). The function of the first two loci has not been characterized. The *Zeo* loci were genetically mapped to the main arm of chromosome 2H. High-resolution genetic mapping of the *Zeo1.b* region assigned it to MLOC_81350 and MLOC_43830 (Houston et al. 2013). The loci encode a transcription factor containing an APETALA2 DNA-binding domain and miR172-binding site in the last exon of MLOC_43830. In mutants of *HvAP2* internode, elongation was reduced in both the culm and the spike, which was the effect of perturbed interaction between miR172 and its corresponding binding site in the mRNA of *AP2*-like transcription factor (Houston et al. 2013). Orthologs reported for rice (Qi et al. 2011) and maize (Jiang et al. 2012) affected internode length as well. The *HvAP2* and *dsp* alleles of barley were included in the discussed group of genes, because they might increase grain yield by controlling spike density, as suggested (Houston et al. 2013; Shahinnia et al. 2012).

Molecular characterization of the Q locus in wheat revealed that it encodes an *AP2*-like transcription factor (Simons et al. 2006). The gene mainly confers the free-threshing character, and is considered as another main domestication gene of polyploid wheat (Dubcovsky and Dvorak 2007).

#### Genes modulating hormone activity

This is another group of genes with a potentially large impact on yield and function in cytokinin and brassinosteroid metabolism and signaling pathways. The genes seem to be of special importance for plant breeding, because both groups of hormones regulate developmental processes and traits directly related to productivity, such as shoot branching, seed number, seed size, root system, and leaf inclination angle.

#### Cytokinins

Cytokinins are crucial in promoting cell division, cell growth and differentiation, axillary bud growth, and in delaying leaf senescence (Jameson and Song 2015; Mok and Mok 2001). This group of plant hormones participates in local and long-distance signaling, interacting with auxins and other substances.

Regulation dependent on cytokinins contributes to crop productivity by controlling cell division and lateral meristem activity (reviewed in Mok and Mok 2001). This growth regulator was shown to be a limiting factor in flower and pod development or during seed setting (Jameson and Song 2015). An important part of the cytokinin regulatory mechanism relies on spatial and temporal changes in cytokinin level. It is precisely controlled in plant tissues and organs. The level of active cytokinins depends on balanced regulation of synthesis [isopentenyl transferase (IPT)], activation [Lonely Guy (LOG)], inactivation (*O*-glucosyl transferase), re-activation (β-glucosidase), and degradation [cytokinin dehydrogenase (CKX)] (Jameson and Song 2015; Sakakibara 2006). The enzyme cytokinin dehydrogenase (CKX) catalyzes cytokinin degradation and is encoded by the family of *CKX* genes (Werner et al. 2006). The expression of individual *CKX* genes, each showing a diverse pattern of spatial and developmental regulation, leads to the desired pattern of cytokinin accumulation in various organs. The significantly higher number of grains observed in an *OsCKX2* knock-out rice mutant was explained to be a result of lower activity of CKX enzyme catalyzing cytokinin degradation and consequently high cytokinin accumulation in the inflorescence meristem (Ashikari et al. 2005). The same effect of increased seed number after RNAi-based silencing of two *HvCKX* genes was reported in barley (Zalewski et al. 2010, 2012, 2014). Decreased expression of *HvCKX1* and *HvCKX9* led to a higher number of seeds and spikes. We proposed that the effect of higher plant productivity is related to the silencing of these *HvCKX* genes, showing high original expression in developing kernels of wild-type plants. The phenotype was significantly stronger in *HvCKX1*-silenced plants compared to *HvCKX9*-silenced ones. This correlated well with higher expression of *HvCKX1* vs. *HvCKX9* in developing kernels of wild-type plants.

In wheat, five haplotype variants of *TaCKX6*-D1 (*a–e*) were evaluated. Haplotype *TaCKX6-D1a* associated with higher grain weight showed decreased expression relative to haplotype *TaCKX6-D1b* in eight DAP seeds (Zhang et al. 2012). Recently, a novel allele of *TaCKX6, TaCKX6a02*, was identified by a PCR-based approach with primers designed for expressed sequence tags (ESTs), which were homoeologous to the *TaCKX* gene family. The allele was associated with grain size, filling rate, and weight (Lu et al. 2015). All these results agree with other data indicating that cytokinins are a key driver of seed yield in various species, as described in the recent review by Jameson and Song (2015).

**Fig. 3:**
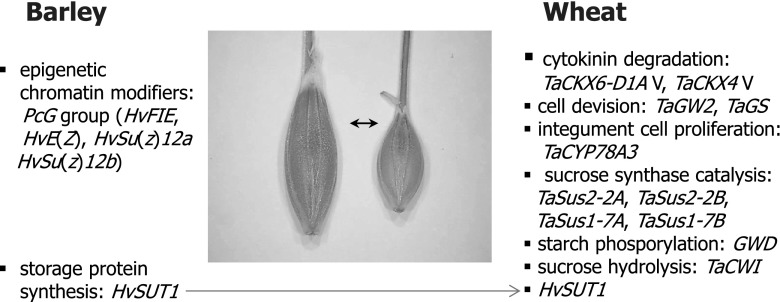
Major genes determining grain size in barley (*left*) and wheat (*right*)

Unexpectedly, an opposite effect was reported for *TaCKX4* variants of recombinant inbred lines (RIL). Grain weight and chlorophyll content in the flag leaf were significantly higher in lines with a suggested higher copy number of the gene (Chang et al. 2015). The authors did not explain how this higher copy number of *TaCKX4* could influence higher grain weight and moreover whether it was correlated with higher expression of the gene. These and others major genes determining grain size in barley and wheat are listed in Fig. 3.

#### Brassinosteroids

Brassinosteroids affect a wide range of physiological processes including cell elongation, cell division and differentiation, reproductive biology (flowering, ovule and seed number, seed size), senescence, root growth and development, activation of photosynthesis, and the antioxidant system (Cao et al. 2005; Che et al. 2015; Houimli et al. 2008; Shahbaz et al. 2008; Wu et al. 2008). Many of the regulated processes are directly associated with crop yield. It is well documented that application of exogenous BR improves plant tolerance for environmental stresses (reviewed in Khripach et al. 2000). Genetic modification of selected BR-related genes led to a crop yield increase by 20–60% (reviewed in Divi and Krishna 2009). Sakamoto et al. (Sakamoto et al. 2006) reported higher grain yield of the rice brassinosteroid-deficient *osdwarf4-1* mutant, characterized by erect leaf phenotype. Increase in the yield, compared with wild-type plants, was evident under conditions of dense planting. According to the authors, the phenotype of more erect leaves improved not only light distribution and photosynthesis but also the size and use of nitrogen reservoirs (Sakamoto et al. 2006).

*uzu1* and *brh* are natural mutants of barley deficient in brassinosteroid biosynthesis. The first one known from the GR period and a big group of *brh* mutants determine reduced stem length. The wild-type *Uzu1* encodes the BR receptor HvBRI1 (BRASSINOSTEROID-INSENSITIVE1; MLOC_5176). The GR mutant allele *uzu1.a* is a monogenic recessive mutant of the brassinosteroid hormone receptor, insensitive to exogenously applied BR. The mutant has been introduced in Japanese hull-less barley cultivars. The *Uzu1* gene is an ortholog of the rice *D61* gene (Chono et al. 2003; Dockter et al. 2014).

Members of another large group of *brh* (*brachytic*) mutants including 27 semidwarf mutants in 18 independent genetic loci (Dahleen et al. 2005) are deficient in brassinosteroid biosynthesis. Their phenotype is very similar to brassinosteroid-deficient mutants. The *brh1, bhr4.j*, and *bhr8.ad* genes have shorter kernels with lower weight compensated by an increased number of kernels per inflorescence and upright plant architecture (Dahleen et al. 2005; Franckowiak and Lundqvist 2012).

Yield-related orthologs of *Uzu1* and *brh* genes in wheat have not been found.

The *GL2* locus in rice, identified as one of the grain-length associated QTLs, has the potential to improve grain weight by up to 27%. It is allelic to *OsGRF4*, which is a component of the BR signaling pathway. Mutation of the gene at the miR369 targeting site results in activation of BR responses and mediates specific regulation of grain length by this group of hormones (Che et al. 2015). Organ specific expression of genes encoding C-22 hydroxylases the enzymes controlling BR levels in rice resulted in plants producing more tillers and more seeds compared with the WT control. The changes led to up to a 44% increase in grain yield per plant (Wu et al. 2008). These two examples of rice genes involved in brassinosteroid signaling or metabolism are included here to indicate the possible huge impact of such genes on yield-related traits. We have suggested searching for their orthologs in wheat and barley.

#### Gibberellins

The GA signaling and metabolism pathways have been prime targets for manipulation for further improvements in crop yield (reviewed in Hedden 2003). The specific genes affecting GA biosynthesis or signaling and used in breeding during GR have been reviewed in the first section. The genes determine the typical phenotype, which is semi-dwarf, with reduced culm internode length and increased lodging resistance.

The widely used semidominant GA insensitive wheat mutant *Rht-1* is an example of a GA signaling mutation. The most important alleles are *Reduced height-1* (*Rht1-B1b, Rht1-D1b*), which in wheat are located in B and D genomes. Additional *Rht-1* dwarfing mutations in B and D genomes were characterized at the molecular level. No semi-dwarfing alleles of *Rht-A1* were found (Pearce et al. 2011). Another *Rht-B1c* allele was partially dominant or co-dominant for plant height, and differed from *Rht-B1a* by a single 2 kb *Veju* retrotransposon insertion, several SNPs and one 197 bp insertion (Wen et al. 2013). Haplotype investigations indicated that retrotransposon insertion was responsible for the extreme dwarfing effect. *RhtB1c* caused reduction of total *Rht-1* transcript levels and up-regulation of GATA-like transcription factors (Wen et al. 2013). The pleiotropic effect of the gene is in this case beneficial for other agricultural traits. It resulted in increased tiller and root number, a higher photosynthetic rate, and a higher chlorophyll content (Zhang et al. 1995; Li et al. 2006). In addition, Rht-B1c determines inhibition of α-amylase synthesis in ripening grains, and enhanced seed dormancy (Flintham and Gale 1982; Flintham et al. 1997). Such a pleiotropic effect, although not always positively associated with higher yield, is typical for the genes involved in plant hormone signaling or metabolism.

Numerous *sdw*-type mutants of barley (Börner et al. 1996; Druka et al. 2011; Franckowiak and Lundqvist 2012; Vu et al. 2010a, b) as well as *HvSLN1* (Wen et al. 2013) represent GA-insensitive phenotype and function in the GA signaling pathway. *HvSLN1* is an ortholog of *Rht1* in wheat and *GAI* in *Arabidopsis*.

Another set of GA-responsive barley mutations function in GA biosynthesis. The wild *Grd5* (*GIBBERELLIN-RESPONSIVE DWARF5*) encodes CYPSSA (cytochrome P450 ent-kaurenoic acid oxidase), the enzyme that catalyzes conversion of ent-kaurenoic acid to GA12, which is the first step of the gibberellin biosynthesis pathway. The three independently obtained *grd5* mutants, defective in CYPSSA enzyme, accumulate ent-kaurenoic acid in developing grains and determine the dwarf phenotype (Helliwell et al. 2001).

*Breviaristatum* (*ari*) represents another group of mutations characterized by semidwarf phenotype. There are 32 *ari* mutant alleles represented in a set of Bowman near-isogenic lines of barley and several hundred at the Nordic Genetic Resource Centre (Dockter and Hansson 2015; Druka et al. 2011; Franckowiak and Lundqvist 2012). The old, widespread cultivated barley cv. Golden Promise carries *ari-e GP* (*erectoides*) recessive mutation (Liu et al. 2014).

Identification and characterization of barley *Polycomb group* (*PcG*) homologs FERTILIZATION INDEPENDENT SEED (*HvFIE*), *ENHANCER OF ZESTE E*(*Z*) [*HvE(Z)*], *SUPPRESSOR OF ZESTE* [*HvSu(z)12a*], and *HvSu(z)12b* revealed that expression of the four genes was significantly different among tissues, developmental stages of seed and barley cultivars. The differences were found to be associated with seed size. Significant differences in *HvFIE* and *HvE(Z)* gene expression affected the level of the abiotic stress-related hormone abscisic acid (ABA), known to be involved in seed maturation, dormancy, and germination (Kapazoglou et al. 2010).

### Other yield-related genes of wheat without known orthologs in barley

Out of 20 yield-related genes characterized in wheat, for only three have the orthologs been reported in barley. There was one gene modifying GA signaling and two representing a group of transcription factors. In addition, two genes encoding enzymes of cytokinin metabolism represent the same family of *CKX* genes that have already been characterized. The 15 other genes with an impact on plant architecture and grain yield (Table S2) include the following: transcription regulators affecting tillering and grain development, and in consequence grain number, regulators of cell divisions mainly affecting grain size, and genes regulating plant architecture by sucrose and starch metabolism.

#### Transcription regulators

In the group of transcription regulators, *Ppd-1* in hexaploid wheat, which is an ortholog of *Ppd-H1* in barley, and *WFZP* in hexaploid wheat as well as *bh* ^*t*^ in tetraploid wheat, which are orthologs of *COM2* in barley, have already been characterized. The transcript elongation factor *TaTEF-7A* is a newly selected gene that represents a multigene family in wheat having a strong impact on grain number (Zheng et al. 2014). The rice ortholog *OsTEF1* was proved to regulate the tillering process by activating expression of cytochrome P450 and regulating expression of another set of over 100 genes (Paul et al. 2012). The wheat *TaTEF-7A* allele, found to increase grain number per spike, was identified as a result of analysis of haplotypes, which showed the highest expression in young spikes and developing seeds. The allele was found to influence grain number per spike. Moreover, ectopic overexpression of *TaTEF-7A* in *A. thaliana* caused a pleiotropic effect on vegetative and reproductive development visible as enhanced grain length, silique number, and silique length (Zheng et al. 2014).

Plant growth and development and consequently grain yield are to a large extent dependent on nitrogen and phosphorus uptake. One component of the uptake system, the trimeric complex Nuclear Factor Y (NF-Y), binds to the CCAAT box, which is a universal element of the eukaryotic promoter. Each of three NF-Y subunits (NF-YA, NF-YB, and NF-YC) is encoded by multiple genes expressed either constitutively or in an organ-specific manner (Stephenson et al. 2007). Many of the genes are involved in the stress response of different species, including cereals (Nelson et al. 2007; Stephenson et al. 2007; Wei et al. 2010). *TaNF-YC11* (Stephenson et al. 2007) and *TaNF-YB3* (Stephenson et al. 2011), two of the light-upregulated NF-YC members in wheat, are involved in the regulation of photosynthesis-related genes. Recently, Yadav et al. (2015) reported that another two, *TaNFYA-B1* and *TaNF-YB4*, play an important role in grain yield. The expression of *TaNFYA-B1* was positively regulated in low nitrogen and phosphorus availability. It was accompanied by up-regulation of both nitrogen and phosphorus uptake transporters in roots and down-regulation of (*tae*)-*miR169*, which in turn can regulate expression of *NFYAs*. The changes of expression also stimulated root development. Overexpression of the gene significantly increased both nitrogen and phosphorus uptake, which allowed for a higher grain yield with lower usage of fertilizers (Qu et al. 2015). The positive role of the second gene, *TaNF-YB4*, in grain yield was documented by introduction of the gene into wheat by biolistic bombardment. Constitutive overexpression in transgenic wheat resulted in development of significantly more spikes and a 20–30% increase in grain yield compared with untransformed control plants under optimal watering conditions (Yadav et al. 2015).

#### Other genes promoting cell division and affecting wheat grain size

Other genes positively affecting grain size participate in various biological processes mainly involved in control of cell expansion and cell division and are not involved in plant hormone regulation.

*GW2* encoding E3 RING ligase was shown to negatively regulate grain size in rice (Song 2007). The enzyme specifically mediates ubiquitination in the ubiquitin-26S proteasome system (Vierstra 2009). Orthologs of the gene have been identified in wheat by means of comparative genomics and cloned. *TaGW2* homologs were found to be present in A, B, and D genomes. *TaGW2-A* and *-D* act in both the cell division and late grain-filling phase, and polymorphism in *TaGW2-A* was associated with grain size (Bednarek et al. 2012; Su et al. 2011). Experimental down-regulation of *TaGW2* by means of RNAi-based silencing gave contradictory results. Plants with suppressed *TaGW2* expression developed lower weight and size grains. Therefore, the gene function was opposite to its wild rice ortholog *OsGW2*, which negatively regulated grain size (Bednarek et al. 2012). Another set of transgenic lines, obtained and characterized by Hong et al. (2014), gave results consistent with the expected function of the gene as a negative regulator of grain size.

Abundance of the *TaGW2* transcript was negatively associated with grain width and weight (Hong et al. 2014). According to Hong et al. (2014), the two sets of conflicting results might be the result of an off-target effect caused by the RNAi cassette in the experiments presented by Bednarek et al. (2012); alternatively, it might be related to the wheat variety used in the experiments. Our experience with RNAi-based silencing in wheat indicates that a very important factor to obtain the silent phenotype without an off-target effect is the transformation method, which might influence the final result (Zalewski et al. 2014). These contradictory results are dispelled in the recent research using the TILLING population, in which the *gw2-A1* mutant allele with G to A transition in the splice acceptor site of exon 5 was identified (Simmonds et al. 2016). The mutation led to mis-splicing in the wild allele *TaGW2-A1*, which resulted in a significant 6.6% increase in thousand-grain weight (TGW) as well as increase of grain width and length in tetraploid and hexaploid wheat.

*Thousand-grain weight6* (*TGW6*) in rice encodes a protein with indole-3-acetic acid (IAA)-glucose hydrolase activity and determines grain weight (Ishimaru et al. 2013). Recently, an ortholog of this gene was found in wheat. Low expression of haplotypes *TaTGW6-b* (InDel mutant) and *TaTGW6-c* (null mutant) was associated with low IAA content and significant increase in grain size and weight (Hanif et al. 2016; Hu et al. 2016). The same comparative genomics approach was used to isolate and characterize *TaGS-D1*, which represents another wheat ortholog of rice *OsGS3* (Zheng et al. 2014). The gene is known to regulate stigma length and stigma extension in rice. *TaGS-D1* was significantly associated with grain weight. InDels found in the introns of the gene in different genotypes influenced higher or lower TGW.

Another cell division promoting grain size *TaGS5* gene in wheat (Ma et al. 2015a) and its ortholog *OsGS5* in rice (Li et al. 2011) were found to play major roles in regulating grain size, purportedly by enhancing the number of cells in both species. The genes encode serine carboxypeptidases (SCPs), which are members of the a/b hydrolase proteins in the S_10 protein family. Homologs of *TaGS5* in wheat, mapped to chromosomes 3 A, 3B, and 3D, were found to be preferentially expressed in young spikes and developing grains. *TaGS5-3A-T* allele, a single nucleotide polymorphism (SNP)-type mutation of *TaGS5-3A*, was shown to be significantly correlated with larger grains and greater thousand kernel weight. This mutation caused overexpression of the gene and significantly higher enzyme activity. An evolutionary approach to analyze the gene in di-, tetra-, and hexaploid wheats revealed that this allele was positively selected in global wheat breeding (Ma et al. 2015a). Rice orthologs of *GS* and *GW* influenced yield mainly through increasing source to sink assimilate flow (Ishimaru et al. 2013).

*TaCYP78A3* is a gene encoding cytochrome P450 CYP78A3. The protein, affecting extension of integument cell proliferation, has been found to be specifically expressed in wheat reproductive organs and positively correlated with the final seed size. Virus-induced gene silencing (VIGS) of the gene in wheat led to a reduced cell number of the seed coat and decreased seed size. Ectopic overexpression of the gene in *A. thaliana* caused 11 to 48% increase of seed size (Ma et al. 2015b).

#### Starch and sucrose metabolism

The genes involved in regulation and metabolic reactions of starch and sucrose as well as nitrogen and phosphorus uptake were shown to affect plant architecture, and thereby grain yield. Starch is the main, up to 70%, component of grain endosperm, so its content can largely account for grain size and weight. Wheat sucrose synthase catalyzing the first step in the conversion of sucrose to starch is encoded by two wheat genes, *TaSus1* and *TaSus2*, which are located in six loci on chromosomes 7A/7B/7D and 2A/2B/2D, respectively (Hou et al. 2014). The differences within the intron sequences between haplotypes were significantly correlated with the differences in thousand-kernel weight (TKW). The favored haplotype containing three SNPs in *TaSus2-2B* was shown to be under strong positive selection in wheat breeding in China (Jiang et al. 2011).

Field trials with transgenic wheat overexpressing barley *HvSUT1* showed increased grain yield and micronutrient concentration (Saalbach et al. 2014; Weichert et al. 2010). The gene encodes the sucrose transporter (SUT), which stimulates storage protein synthesis. Another yield-related wheat anther-specific invertase gene, *TaCWI*, encodes cell wall invertase (CWI), which hydrolyzes sucrose into glucose and fructose. Among several haplotypes tested, only one, *TaCWI*-*5D*, was significantly associated with TKW (Jiang et al. 2015).

Glucan, water dikinase (GWD) is the primary enzyme required for starch phosphorylation. RNAi-mediated down-regulation of the gene, controlled by an endosperm-specific promoter, led to decreased GWD activity and starch phosphate content as well as several pleiotropic effects: higher α-amylase activity in the aleurone layer in mature grains as well as unexpected elevated grain size, early vigor and plant biomass (Ral et al. 2012; Corrigendum 2013).

The mutant of tiller inhibition, *tin*, opposite to the above-described mutants, is a negative example of grain productivity. The reduced tillering observed in the *tin* mutant is the result of inhibition of tiller bud outgrowth during the shoot apex transition from the vegetative to reproductive stage (Kebrom et al. 2012). The authors hypothesize that the *tin* mutant is associated with precocious internode development that diverts sucrose from developing tillers.

### Conclusions

The potential annual yield increase of wheat achieved as a result of breeding is estimated at about 0.5% (Fischer et al. 2009). Furthermore, the globally observed multiyear tendency of yield growth is decreasing. These observations contrast starkly with the anticipated growth of the population, food demand, and bio-based fuel production. We propose that improving cereal productivity will need to use current knowledge on yield-determining genes in breeding programs incorporating these genes into the best yielding cultivars. This strategy combining current knowledge gained from modern biotechnology and at the same time based on natural mutants could be a creative continuation of the GR approach, which proved to be so successful.

As shown here, it is very important that genes identified as potential targets for yield-focused selection should be characterized for their function and interaction with other genes. It is crucial, because only certain allele combinations are beneficial for yield. Biotechnology creates the possibilities to modify a selected gene or to alter the gene’s expression to reveal the function of the gene. These approaches are particularly important for polyploid wheat, where natural mutants are rarely available. RNAi-based gene silencing allows down-regulation of a single selected gene, several homoeologs or a group of genes belonging to a gene family. This approach, relatively straightforward for loss of function gene analysis, generates transgenic lines with diverse silencing ratios. This opens the possibility for functional analysis of a gene, whose knock-out mutation is lethal or semi-lethal. The rapidly developing gene-editing CRISPR/Cas system is already considered a breakthrough technology. It offers the possibility to change the selected region of a targeted gene, which leads to knock-out mutation (the easiest to achieve) or alternatively to specific up- or down-regulation of gene expression. Since this can be done already in the selected cultivar, it alleviates the need for lengthy backcrossing. It is important to add that gene editing is indistinguishable from natural mutations. At this point, it is not decided whether this technology will be excluded in the EU from GMO legislature; however, the first FDA decisions on CRISPR/Cas-edited champignons confirmed non-GMO status of the plant. Combining rapidly developing CRISPR/Cas systems with a knowledge of gene function could speed up the process of creating new, better yielding cultivars. Moreover, the knowledge of a specific allele function and its nucleotide sequence should be used to identify natural mutants or haplotypes in a natural population or induced mutants using the TILLING strategy. This also enables the design of allele specific markers for efficient phenotype/haplotype selection during breeding.

The implementation of selected genes in breeding programs requires consideration of specific genotypes, agronomic, and climate conditions and the fact that many of the genes are members of multigene families. Modulated expression of flowering genes, which regulate photoperiod and vernalization-dependent floral induction, might be advantageous for spring or winter varieties under long-day or short-day conditions. Very spectacular yield effects were reported for genes involved in hormone regulation, gibberellins, brassinosteroids, and cytokinins. Since genes of gibberellin and brassinosteroid metabolism and signaling are mainly involved in culm robustness, the cytokinin metabolism genes have a big impact on grain number in barley and rice and grain size in wheat. These organ-specific genes regulate cytokinin homeostasis within reproductive organs, and they are mostly independent of agronomic or climate conditions. The general rule is that reduced expression of *CKX* in specified organs leads to increased yield. However, their regulation may depend on whether the crop is sink limited or grows in a source-limiting environment. The source/sink regulation might be coordinated with the sucrose and starch metabolism genes increasing grain size in wheat as well as the yield-increasing, nitrate signaling *NAC* and nitrogen and phosphorus usage *NFY* genes.

Many yield-related traits and hormone regulators are encoded by multigene families. Their expression patterns, showing strong spatial and temporal regulation, provide strong indications for their possible role as yield-affecting components. Thus, transcriptome analysis could be the first step of screening the candidate genes. Special attention should be paid to microRNA encoding genes. It is estimated that one-third of all protein encoding genes are regulated by microRNAs (Jones-Rhoades et al. 2006), which complies with the increasing number of reports proving that these regulators are crucial in cereal productivity.

All characterized genes determine various growth components, which should be combined with nutritional status, the activity of particular phytohormones, and the synchronization of floret development. Pyramiding of these yield-affecting genes with other agronomically important genes might be proposed for further improvement.

#### Author contribution statement

ANO is the main author of the concept and the design of the review, including supplementary tables. IR is the author of Figs. 1, 2 and 3, and participated in preparing the literature. WO took part in designing, writing and discussion. SG contributed to the discussion and preparation of supplementary tables.

## Electronic supplementary material

Below is the link to the electronic supplementary material.

Supplementary material 1 (PDF 74 KB)

Supplementary material 2 (PDF 74 KB)
