# Supplementary material for: Major genes determining yield-related traits in wheat and barley
Source: Theor Appl Genet. 2017 Mar 17;130(6):1081–98. doi: 10.1007/s00122-017-2880-x (PMC5440550; doi:10.1007/s00122-017-2880-x)
Supplement: Supplementary file 1 — Supplementary material 1 (PDF 74 KB) [file 122_2017_2880_MOESM1_ESM.pdf]

Table S1. Main genes of barley (*Hordeum vulgare*) determining yield components.

Approach / locus	Locus/gene/ protein class	Function/orthologs	Modifiers/regulators	Affected yield components	References
**Transcription factors and other regulators – spikelet development, inflorescence architecture and growth – grain number**					
Mutant	*Vrs1* (*SIX-ROWED SPIKE 1*); a homeodomain-leucine zipper I-class (HD-Zip I) type transcription factor	Cell division and development in the lateral spikelets; no orthologs	*Vrs1.b* – wild type allele, *VRS1* – loss of function mutant; modifiers – *INT* alleles (see below)	*VRS1* – six-row spikes, reduction in the number of tillers<br>*Vrs1.b* – two-rowed spikes	(Komatsuda et al. 2007; Lundqvist et al. 1997; Ramsay et al. 2011)
Mutant	*INT* (*INTERMEDIUM*); HD-ZIP I-class homeobox transcription factor	Modifier of lateral spikelet fertility; orthologs: maize *TEOSINTE BRANCHED 1* (*TB1*) and barley *Photoperiod-1* (*Ppd-H1*)	*INT-C* alleles – quantitative variation; *vrs1.a* in six-rowed (loss of function allele) is complemented by *Int-c.a* allele and in two-rowed *Vrs1.b* is complemented by *int-c.b*	Variations in male fertility and grain development from two-rowed to six-rowed	(Lundqvist et al. 1997; Lundqvist and Lundqvist 1988; Ramsay et al. 2011)
Mutant, positional cloning	*Ppd-H1 (Photoperiod-1)*, a member of pseudo-response regulator (*PPR*) family genes	The major determinant of photoperiod response; ortholog in wheat	*ppd-H1* mutant, reduced photoperiod responsiveness altered circadian expression of the photoperiod pathway gene *CONSTANS* and reduced expression of its downstream target, *FT*	Induction of flowering, highly advantageous in spring-sown varieties	(Turner et al. 2005)
Natural mutation, positional cloning, gene isolation	*com2* (*compositum 2*); AP2/ERF transcription factors	Branch repression pathway; orthologs in tetraploid and hexaploid wheat	*COM2* expression is regulated by *Six-rowed spike 4* influencing branch repression; *HvIDS1* is a putative down-stream target of *COM2*	Spikelets are replaced by lateral secondary spikes; significantly more grains per spike, higher spike yield	(Poursarebani et al. 2015; Sharman 1994)

Mapping MLOC_43830	*HvAP2* (*APETALA 2*); transcription factor two, AP2 DNA-binding domain and miR172-binding site in its last exon	Perturbed interaction between miR172 and its corresponding binding site in the mRNA of *AP2* like transcription factor; orthologs in rice, wheat and maize	Mutant alleles – internode elongation is reduced in both the culm and the spike	A key controller of barley spike density	(Houston et al. 2013)
DatabasesMLOC_65101.1	*HvNAC005*; NAC-a6 transcription factor	Positive regulator of senescence	Over-expression – delayed development combined with precocious senescence; up regulation of genes involved in secondary metabolism, hormone metabolism, stress, signaling, development and transport	Nutrient remobilization related to senescence process	(Christiansen et al. 2016; 2011)
Mutant, mapping and cloning by sequencing	*Blade-On-Petiole 1 (BOP1)* and *BOP2*; putative transcriptional coactivator in *A. thaliana*	Developmental growth of vegetative and reproductive organs; partially conserved function between *Arabidopsis* and barley	*BOP1*/2 controls internode length and causes pleiotropic changes in spike development; haplotype diversity	Internode length, spike development	(Jost et al. 2016)
Natural mutations, mapping	*dsp* (*dense spike*)	Controlling the barley spike and spikelet characteristics	28 alleles	A reduction in length of rachis internodes, shorter culms, compact spikes, smaller volume of dew (reduced risk of fungal infections and pre-harvest sprouting)	(Shahinnia et al. 2012)
Spontaneous mutation	*ert-a* (*erectoides-a*)	A second dense spike locus	43 alleles,<br>*ert-a.6* shorter plants and kernels, reduced grain yield	Resistance to lodging, semi-compact spikes with dense basis, reduced culm length, improved culm strength, susceptibility to powdery mildew, virus and nematode diseases	(Druka et al. 2011; Franckowiak and Lundqvist 2012) (mapping)

Induced mutations, mapping	*ert-k.32* (*erectoides-k.32*)	Controlling culm length and spike architecture	*ert-k.32* – induced, approved as a cultivar; eight allelic *ert-k* mutants	Resistance to lodging; alternative to pleiotropic GR alleles of the *Sdw1* and *Uzu1*	(Franckowiak and Lundqvist 2012; Kristensen et al. 2016)
**Genes of cytokinin metabolism – plant architecture – grain number**					
RNAi	*HvCKX1* cytokinin dehydrogenase enzyme HvCKX1	CKX enzymes irreversibly degrade cytokinins, temporal and spatial control of cytokinin level; orthologs in wheat and other species	Multigene family<br>Decreased expression of the *HvCKX1* gene by RNAi silencing and CKX enzyme in the developing spikes	Higher number of seeds and spikes	(Zalewski et al. 2010)
RNAi	*HvCKX9* cytokinin dehydrogenase enzyme HvCKX9	As above	One of multigene family<br>Decreased expression of the *HvCKX9* gene by RNAi silencing and CKX enzyme in the developing spikes	Higher number of seeds and spikes	(Zalewski et al. 2014; Zalewski et al. 2012)
**Gibberellin signaling and metabolism – plant architecture – culm (stem) robustness/hardiness**					
Natural or induced mutations	*Sdw* (*semidwarf*); *Sdw1/Denso* locus	GR gene, GA-insensitive, GA signaling?	53 mutants; numerous different mother cultivars<br>*Sdw1/Denso* locus – first reported allele *sdw1.c*; second allele – *sdw1.a*; third *sdw1.d* the most important – main allele in modern European; fourth *sdw1.e*	Semi-dwarf, reduction of culm and rachis internode length and increased lodging resistance, reduced grain size	(Druka et al. 2011; Franckowiak and Lundqvist 2012)
Mutant Hv287<br>Map based cloning	*sdw3 (semidwarf 3)* former *gai*	GA-insensitive, GA signaling	Monogenic recessive trait; lack of gene identity (unidentified)	Mild semi-dwarf, lodging-resistant	(Börner et al. 1999; Vu et al. 2010)
Mutant	*sdw4 (semidwarf 4)*	GA-insensitive, GA signaling	*sdw4.ba* – recessive, short-culm mutant with enlarged flag leaves	semi-dwarf, enhanced lodging-resistant; enlarged flag leaf	(Franckowiak and Lundqvist 2012)
Natural mutants	*HvSln1* (*Slender1*) – mutation in DELLA proteins	GA-insensitive, GA signaling; ortholog of *Rht1* in wheat and *GAI* in	*sln1b* allele – single amino acid substitution in DELLA domain, recessive loss of function alleles	Pleiotropic effects: semi-dwarf, slender-type plant architecture	(Chandler et al. 2002; Wen et al. 2013)

		Arabidopsis	(translational stop codon)		
Mutants	*Grd5* (*GIBBERELLIN-RESPONSIVE DWARF*), CYPSSA – cytochrome P450 *ent*-kaurenoic acid oxidase	Involved in GA metabolism, the enzyme catalyzes the first step of the gibberellin biosynthetic pathway from *ent*-kaurenoic acid to GA12; ortholog in maize	*grd5* – gibberellin-responsive, three independent mutants contain mutations in a gene encoding a member of the CYP88A subfamily of cytochrome P450 enzymes,	Dwarfing	(Helliwell et al. 2001)
**Brassinosteroid (BR) signaling and metabolism – plant architecture – culm length**					
Natural mutation	*Uzu1* (MLOC_5176; *UZU DWARF*) brassinosteroid hormone receptor BRASSINOSTEROID-INSENSITIVE HvBRI1	GR gene, BR insensitive, deficient in brassinosteroid biosynthesis; ortholog of the rice *D61*	Mutation in the brassinosteroid receptor causing an His-857 to Arg exchange in the kinase domain of the receptor<br>*uzu1.a* allele – monogenic recessive mutation	Short-culm in six-rowed barley in East Asia, highly resistant to lodging, upright plant architecture, low leaf angles (high photosynthetic efficiency; facilitates dense crop planting, heat sensitive)	(Chono et al. 2003; Dockter et al. 2014)
Mutants	*brh (brachytic)*	Deficient in brassinosteroid biosynthesis	A group of mutants (27 semidwarf mutants in 18 independent genetic loci, e.g. *brh1*, *bhr4*.j, *bhr8.ad*)	Reduced culm length, shorter kernels with lower weight but with an increased number of kernels per inflorescence, upright plant architecture	(Dahleen et al. 2005; Franckowiak and Lundqvist 2012)
**Other – plant architecture (culm and awn length), grain size**					
Mutants	*ari (breviaristatum)*<br>*ari-e GP* (*erectoides* in cv. Golden Promise)	Semi dwarfing mutant	32 *ari* mutant alleles in Bowman near-isogenic lines, several hundred at the Nordic Genetic Resource Centre<br>*ari-e* recessive mutant – Golden Promise (Liu et al. 2014)	Reduced awn length, a number of mutants have shortened culm, and upright arch	(Druka et al. 2011; Franckowiak and Lundqvist 2012)
Homologs	*PcG* Polycomb group protein complexes (PcG)	Involved in seed development and in response to external ABA, ultimately leading to	Four homologs: *HvFIE*, *HvE(Z)*, *HvSu(z)12a*, and *HvSu(z)12b*. Gene expression is responsive to abscisic acid (ABA).	Seed size	(Kapazoglou et al. 2010)

		chromatin compaction and gene silencing			
