# Supplementary material for: Major genes determining yield-related traits in wheat and barley
Source: Theor Appl Genet. 2017 Mar 17;130(6):1081–98. doi: 10.1007/s00122-017-2880-x (PMC5440550; doi:10.1007/s00122-017-2880-x)
Supplement: Supplementary file 2 — Supplementary material 2 (PDF 74 KB) [file 122_2017_2880_MOESM2_ESM.pdf]

Table S2. Main genes of wheat (*Triticum aestivum* L.) determining yield components.

Approach / locus	Gene/protein class	Function/Orthologs	Alleles/Modifiers/Regulators	Affected yield components	References
**Transcription regulators (floral) – inflorescence architecture – grain number**					
QTL, mutant analyses *FT FLOWERING LOCUS*	*Ppd-D1a* (*Photoperiod-1*) a member of the pseudo-response regulator gene family (PPR)	Controls photoperiod-dependent floral induction; regulates inflorescence architecture and paired spikelet development by modulating the strength of the floral promoting signal (*FT*); ortholog in barley	*Ppd-1* – major inhibitory effect on paired spikelet formation by regulating the expression of *FT* *Ppd-D1a* loss of function allele – photoperiod-insensitive, promotes constitutive LD response	Temperate cereals – allelic variation in *Ppd-1* influences sensitivity to long-day conditions. Modulated expression of *Ppd*-1 and *FT* can be used to form a wheat inflorescence with a better arrangement and increased number of grains in spikelets	(Beales et al. 2007; Boden et al. 2015; Li et al. 2011; Shaw et al. 2013; Shaw et al. 2012; Turner et al. 2005)
Natural mutants	*FRIZZY PANICLE* (*WFZP*; *T. aestivum*), *branched head*^{t} (*bh*^{t} *T. turgidum*) APETALA2/ Ethylene Response Factor	A key player in spikelet development during the floret meristem transition phase; non-canonical spike-branching; orthologs in barley, rice, maize	Three *FZP* homologous genes *WFZP* – recessive, coding mutations of *WFZP-D* – cause the SS phenotype, the most severe in *FZP-D* lesions + *FZP-A* frameshift mutants	Multirow spike (MRS) in hexaploid wheat and tetraploid 'Miracle-Wheat', significantly more grains per spike, higher spike yield	(Dobrovolskaya et al. 2015; Poursarebani et al. 2015)
Haplotypes, NILs, gene isolation, overexpression in *A. thaliana*	*TaTEF-7A*; TaTEF-7A transcript elongation factor	*OsTEF1* regulates the tillering process by inducing expression of cytochrome P450	*TaTEF-7A* allele – the highest expression in young spikes and developing seeds; overexpression in *A. thaliana*	Increased grain number, enhance grain length, silique number and silique length	(Paul et al. 2012; Zheng et al. 2014)
Genome identification, gene cloning, phylogenetic	*NFYAs*, *NFYBs*, *NFYCs* Nuclear Factor Y (NF-Y): subunits NF-YA, NF-YB, NF-YC	Essential role in root development and nitrogen and phosphorus usage; orthologs in maize (*ZmNF-YB2*) and other species	Each of the NF-Y subunits is encoded by multiple genes (18 *NFYAs*, 34 *NFYBs*, 28 *NFYCs*). *TaNFYA-B1* is a low nitrogen and low phosphorus-inducible gene –	Increased grain yield and root development under control, low N and low P conditions, high yield with less fertilizer input	(Qu et al. 2015; Yadav et al. 2015)

analysis, transgenic overexpression of *TaNF-YB4*	that bind to the CCAAT box, a ubiquitous eukaryotic promoter element		increase in nitrogen and phosphorus uptake, upregulation of the expression of both nitrate and phosphate transporters in roots + down-regulation of miR169	Overexpression stimulates lateral branching. Transgenics increased grain yield by 20–30%.	
QTL *Gpc-B1*, cloning, RNAi	*NAM-B1* NAC transcription factor	*NAC* family genes regulate NAC protein level and localization and participate in transcription complex including microRNA: specific to plants	NAC accelerates senescence and improves grain protein, zinc and iron content; RNA reduction delayed senescence	Grain size	(Uauy et al. 2006 a,b; rev. function Olsen et al. 2005)
Gene isolation, overexpression in transgenic wheat lines	*TaNAC2*-5A – a nitrate inducible and cereal-specific NAM, ATAF, and CUC (NAC) transcription factor	TaNAC2-5A binds to the promoter regions of the genes encoding nitrate transporter and glutamine synthetase; involved in nitrate signaling; members in other species	Overexpression of *TaNAC2-5A* in wheat enhanced root growth and nitrate influx rate	Higher grain yield and higher nitrogen accumulation in aerial parts and allocated more nitrogen to grains in a field experiment	(He et al. 2015)
RNAi	*TaABCC13*; TaABCC13 functional transporter	Functionally important for grain development and detoxification of heavy metals.	Silencing of *TaABCC13* reduced PA in mature grains and developmental defects	Grain development	(Bhati et al. 2016)
**Genes of cytokinin metabolism – plant architecture – grain size**					
Haplotype variants	*TaCKX6-D1*; CKX – cytokinin dehydrogenase	CKX enzymes irreversibly degrade cytokinins; temporal and spatial control of cytokinin level; orthologs in barley, rice (*OsCKX2*) and family members in other species	Five haplotypes *a-e*. Haplotype *TaCKX6-D1a* associated with higher grain weight – decreased expression relative to haplo b in 8 DAP seeds; novel allele *TaCKX6a02*	Grain weight, grain size, filling rate	(Lu et al. 2015; Zhang et al. 2012)
Recombinant inbred lines (RIL) QTL	*TaCKX4*; CKX - cytokinin dehydrogenase	As above	Significant association of copy number variation of *TaCKX4* with flag leaf chlorophyll content after anthesis and grain weight	Grain weight	(Chang et al. 2015)
**Gibberellin signaling – plant architecture – culm (stem) robustness/hardiness**					

Natural mutations	*Rht-B1* and *Rht-D1*, *Rht-B1c* DELLA proteins that share an N-terminal DELLA domain	GR genes, transcriptional regulators that negatively regulate GA signaling, suggested regulation of GATA-like transcription factor; ortholog of *Arabidopsis GAI* and *SLN1* in barley	GA insensitive alleles of the *Rht-B1* and *Rht-D1* loci; semidominant mutations (more than 20); truncated proteins lacking the DELLA motif cannot respond to GA3; *Rht-B1c* partially dominant or co-dominant for plant height, differed from *Rht-B1a* by one 2 kb *Veju* retrotransposon insertion, primarily responsible for the extreme dwarfing effect	Reduced plant height and a compact lodging-resistance phenotype, effect on seed dormancy, and photosynthesis	(Pearce et al. 2011; Peng et al. 1999; Sun and Gubler 2004; Wen et al. 2013)
Natural mutation, RIL, fine mapping	*Rht8*; as above	Reduced cell elongation; reduced sensitivity to brassinosteroids	Not known	Shorter intermodal segments and culm, improved lodging resistance; used in dry environments	(Gasperini et al. 2012)
**Cell division/proliferation regulators – grain size**					
QTL – rice, comparative genomics, cloning, TILLING	*TaGW2* (*GRAIN WIDTH 2*) *OsGW2* encodes an E3 RING ligase	*TaGW2-A* and *-D* act in both the cell division and late grain-filling phase; ortholog in rice	Homologs in the A, B, D genomes *TaGW2* – down-regulation, significant reduction in endosperm cell number; *gw2-A1* – significant increase of grain size and weight	Grain size and weight	(Bednarek et al. 2012; Hong et al. 2014; Simmonds et al. 2016; Su et al. 2011)
Comparative genomics, haplotypes	*TaTGW6* indole-3-acetic acid-glucose hydrolase	Low expression of *TaTGW6* is associated with low IAA content and high grain weight; ortholog in rice (*TGW6*)	*TaTGW6-a*, *TaTGW6-A1b* – low TGW; *TaTGW6-A1a* (3 SNPs), *TaTGW6-b* (InDel mutant), *TaTGW6-c* (null mutant) – last two low or lack of expression and significant increase in grain size and weight	Thousand grain weight (TGW) and yield	(Hanif et al. 2016; Hu et al. 2016)
Comparative genomics	*TaGS-D1*	Regulate stigma length and stigma extension in rice; ortholog of rice *OsGS3*	InDels in the introns of the gene between genotypes – higher or lower TGW	Grain weight and grain length	(Zhang et al. 2014)
VIGS	*TaCYP78A3*	Affects the extent of	Specifically expressed in wheat	Grain size: 11–48%	(Ma et al.

silencing, overexpression (*A. thaliana*)	cytochrome P450 CYP78A3 protein	integument cell proliferation	reproductive organs; positively correlated with the final seed size	increase in *Arabidopsis* seed size	2015b)
Comparative genomics, transgenic rice	*TaGS5-3A-T*, serine carboxypeptidases (SCPs), members of the a/b hydrolase proteins in the S_10 protein family	Promotes cell division by regulating cell cycle genes resulting in large grain size generated by an increased cell number; ortholog in rice *OsGS5*	*TaGS5-3A-T* preferentially expressed in young spikes and developing grains, significantly correlated with larger grain size and higher thousand kernel weight	Larger grain size, larger kernel and yield	(Ma et al. 2015a)
**Sucrose, starch metabolism – plant architecture – grain yield**					
Transgenic wheat, field trials	*HvSUT1*, sucrose transporter (SUT)	Stimulates storage protein synthesis	Up-regulated gene expression of positive and negative regulators of sugar signaling and assimilate supply	Increased grain yield and micronutrient concentration	(Saalbach et al. 2014; Weichert et al. 2010)
Comparative genomics, haplotypes	*TaSus1* and *TaSus2* sucrose synthase SUS	Sucrose synthase catalysis is the first step in the conversion of sucrose to starch	Loci: *TaSus2-2A, TaSus2-2B, TaSus1-7A*, and *TaSus1-7B* main variations within the introns; significant differences between the haplotypes correlated with TKW differences	Larger grain size	(Hou et al. 2014)
Haplotypes	*TaCWI*, cell wall invertase (CWI),	Wheat anther-specific invertase genes; hydrolyzes sucrose into glucose and fructose; orthologs in rice	*TaCWI-4A, TaCWI-5D* – SNPs *TaCWI-5B* – no polymorphism; *TaCWI-5D* significantly associated with TKW	Higher thousand kernel weight	(Jiang et al. 2015)
Natural mutant	*T6P/SnRK1* trehalose6-phosphate (T6P)	T6P is the intermediary in TRE synthesis formed from glucose-6-phosphate and UDP-glucose; source/sink interaction	Not known	Expected function to determine grain yield	(reviewed by Lawlor and Paul 2014)
RNAi	*GWD* GWD – glucan, water-dikinase, the primary enzyme required for starch	unclear	RNAi-mediated down-regulation (endosperm-specific promoter) – a decrease in starch phosphate content and an increase in grain size (+ increased level of α-amylase activity	Increases in grain size, early vigor and plant biomass	(Ral et al. 2013; Ral et al. 2012)

	phosphorylation		in the aleurone layer during grain maturation)		
Induced mutant	*Tin* (tiller inhibition)	Reduced tillering due to early cessation of tiller bud outgrowth during the transition of the shoot apex from the vegetative to the reproductive stage.	Upregulation of a gene induced by Suc starvation, downregulation of a *Suc-inducible* gene, and reduced Suc content in dormant tin buds	Reduced tillering (branching)	(Kebrom et al. 2012)
